# Dental Mesenchymal Stem/Stromal Cells and Their Exosomes

**DOI:** 10.1155/2018/8973613

**Published:** 2018-04-15

**Authors:** Peter Stanko, Ursula Altanerova, Jana Jakubechova, Vanda Repiska, Cestmir Altaner

**Affiliations:** ^1^Department of Stomatology and Maxillofacial Surgery, Faculty of Medicine, Comenius University in Bratislava, Bratislava, Slovakia; ^2^Department of Stem Cell Preparation, St. Elisabeth Cancer Institute, Bratislava, Slovakia; ^3^Institute of Medical Biology, Genetics and Clinical Genetics, Faculty of Medicine, Comenius University in Bratislava, Bratislava, Slovakia; ^4^Cancer Research Institute, BMC, Slovak Academy of Sciences, Bratislava, Slovakia

## Abstract

Stem cells derived from human dental pulp tissue (DP-MSC) differ from the other mesenchymal stem cells prepared from bone marrow or adipose tissue due to their embryonic origin from the neural crest and are of special interest because of their neurotropic character. Furthermore, the therapeutic potential of DP-MSCs is realized through paracrine action of extracellularly released components, for which exosomes play an important role. In this review, we intend to explore the properties of these cells with an emphasis on exosomes. The therapeutic applicability of these cells and exosomes in dental practice, neurodegenerative diseases, and many other difficultly treatable diseases, like myocardial infarction, focal cerebral ischemia, acute lung or brain injury, acute respiratory distress syndrome, acute inflammation, and several others is concisely covered. The use of cellular exosomes as an important diagnostic marker and indicator of targeted cancer therapies is also discussed, while the importance of stem cells from human exfoliated deciduous teeth as a source of evolutionally young cells for future regenerative therapies is stressed. We conclude that exosomes derived from these cells are potent therapeutic tools for regenerative medicine in the near future as clinical administration of DP-MSC-conditioned medium and/or exosomes is safer and more practical than stem cells.

## 1. Introduction

The involvement of mesenchymal stem cells (MSCs) in the regeneration of damaged or aged tissues is well supported by current research. Furthermore, cell manufacturing procedures for obtaining high quality, bioactive MSCs from human bone marrow has been approved by the US Food and Drug Administration [1], while recent developments in regenerative medicine have suggested that there is a paracrine/endocrine mechanism involved with MSC-mediated repair of damaged tissues. Initially, as Pittenger and colleagues pointed out, MSCs were used primarily for their cytokine and growth factor production rather than for their cell replacement and differentiation ability [2]. Later on, it was recognized that the most effective contribution to the regenerative process comes from exosomes released from MSCs. These exosomes have a complex composition that mirrors not only their parental cells but also their ability to migrate towards specific tissue [3]. This property is common for MSCs regardless of their tissue origin.

Dental pulp mesenchymal stem/stromal cells (DP-MSCs) are of particular interest because of their neurotropic character, which makes DP-MSCs and their exosomes particularly attractive as a new therapeutic tool for the alleviation of symptoms of neurodegenerative diseases and many other difficultly treatable maladies. Dental tissue-derived stem cells besides DP-MSCs include multiple types such as stem cells from exfoliated deciduous teeth (SHED), stem cells from apical papilla (SCAP), periodontal ligament stem cells (PDLSCs), and dental follicle progenitor cells (DFPCs) [4]. All of them can be isolated from a single tooth and behave as mesenchymal stem/stromal cells. MSCs derived from different dental tissues possess multiple differentiation capabilities. In vitro comparisons of the properties of different types of human dental MSCs, such as their multipotentiality and other phenotypic characteristics, have been performed and comprehensively reviewed [5]. From the regenerative medicine point of view, the most valuable cells are the deciduous tooth cells, which, being young, are nearest to embryonic character and distinguishable from stem cells isolated from adult teeth. However, despite their differences, all of these dental tissue-derived stem cells are not distinguishable morphologically and do not differ in a statistically significant way in their secreted components.

Quantitative gene expression analysis of MSCs isolated from the umbilical cord (UC) and dental pulp (DP) generally reflects their biological functions. Genes related to cell proliferation, angiogenesis, and immune responses are expressed at higher levels in UC, whereas genes related to growth factors, receptor activity, and signal transduction are more highly expressed in DP [6]. MSCs are called stromal, mesenchymal, or medical signaling cells depending on their biological functions [7, 8]. However, whether all these cell populations differ in the quality of secretomes or in the composition of exosome cargo as a driving force of their regenerative action remains to be determined.

## 2. Dental Pulp Mesenchymal Stem/Stromal Cells

**Figure 1:**
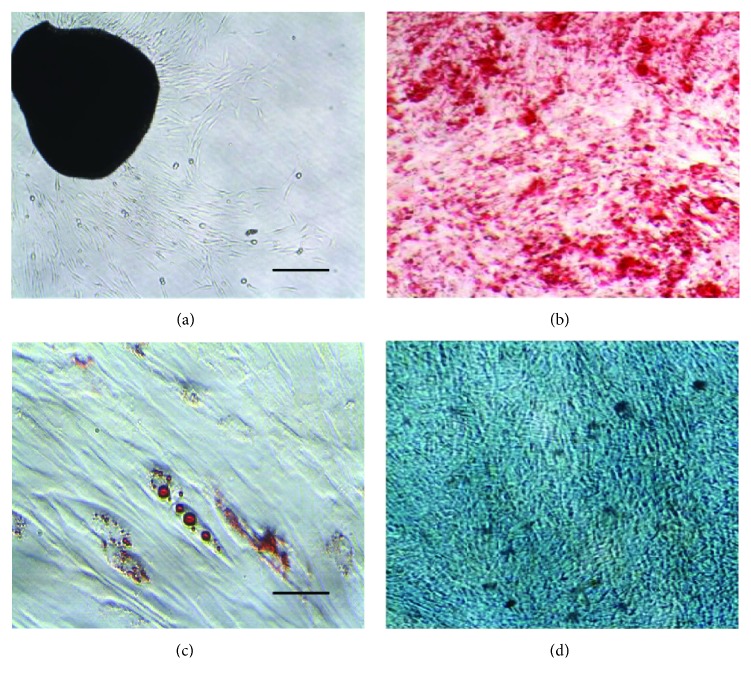
Growth of DP-MSCs from explants and their differentiation. (a) DP-MSCs growing from explants of dental pond tissue of the deciduous tooth. (b) Differentiation into osteoblasts (stained with Alizarin Red S). (c) Differentiation into adipocytes (stained with Oil Red O). (d) Differentiation into chondrocytes (stained with Alcian blue). Scale bars = 100 *μ*m.

DP-MSCs are known for their high proliferative potential, as demonstrated by their ability to be isolated and expanded from dental pulp tissue fragments that adhere to plastic tissue culture dishes. These tissue fragments can be transferred from one dish to another for 3 months with no interruption in cell proliferation. The cells outgrowing from the explants and the differentiation of DP-MSCs to three lineages in vitro are depicted in Figure 1. In agreement with a recent report [9], we found extract of human platelets to be an optimal growth supplement for the expansion of DP-MSCs for clinical scale manufacturing. Additionally, DP-MSCs do not differ substantively from MSCs derived from adipose tissue, bone marrow, and umbilical cord tissue with regard to the internationally accepted vague criteria of plastic adherence and the ability to differentiate into osteoblasts, chondrocytes, and adipocytes in vitro while expressing mesenchymal (CD29, CD90, CD105, CD73, and CD44) but not hematopoietic lineage markers (CD14, CD34, and CD45) [10].

## 3. Embryonic Origin of DP-MSCs

DP-MSCs have been intensively studied because the dental pulp is made of ecto-mesenchymal elements, categorizing DP-MSCs as neural crest-derived cells. Specifically, DP-MSCs from the dental pulp of third molar teeth or human stem cells from exfoliated SHEDs have unique neurogenic properties that could potentially be exploited for therapeutic use. Our comparison of DP-MSCs with bone marrow and adipose tissue-derived MSCs (AT-MSCs) revealed differences in the expression of pluripotent stem cell genes that reflect the ecto-mesenchymal origin of DP-MSCs [11]. DP-MSCs are heterologous, as isolated cell clones differ in both proliferative rates and the ability to differentiate along various lineages. Furthermore, proliferative cell populations possess longer telomeres than less proliferative populations [12]. The neurologic tropism of human DP-MSCs was reflected in the finding that these cells, labelled with iron oxide nanoparticles, internalized to the rat brain after intranasal application [13]. Furthermore, it has been reported that adult dental pulp cells isolated from third molars have the capability to differentiate into keratocytes, the cells of the corneal stroma. While after inducing differentiation in vitro, the cells expressed molecules characteristic of keratocytes, keratocan, and keratan sulfate proteoglycans at both the gene and the protein levels [14].

## 4. Odontogenic Cells for Regenerative Dental Therapies

The dental-tissue-derived stem cells are isolated from specialized tissue and therefore could have the potential to differentiate into odontogenic cells. Out of all of them, DP-MSCs show the greatest potential to produce a comparatively high volume of mineralized matrix during in vitro experiments. However, Hung along with collaborators demonstrated that both implants of AT-MSCs and DP-MSCs were able to grow self-assembled, new teeth in adult rabbit extraction sockets with a high success rate. Furthermore, both dentinogenesis and mineral volume was enhanced when using AT-MSCs, suggesting that they might also be useful for regenerative dental therapies [15]. The reaserchers found that the difficulty normally found in obtaining sufficient amounts of dental stem cells was bypassed by the usage of more accessible MSCs like AT-MSCs and bone marrow-derived MSCs (BM-MSCs). It has been recognized that upon incubation of these cells in the conditioned media (CM) from dental pulp stem cell-derived osteoblasts and auricle cartilage chondrocytes, these cells develop the capability of differentiating into osteoblast and chondroblast lineages [16]. Additionally, the expression of proangiogenic factors was achieved by coimplantation of MSCs with endothelial cells, accelerated pulp tissue, and dentin regeneration in a rat model of molar coronal pulp regeneration [17].

Also, preclinical studies of the efficacy of stem cells on periodontal regeneration were recently comprehensively reviewed [18]. In an evaluation of the promotion of periodontal regeneration, the variety of dental tissue origin MSCs, both autologous and allogeneic, as well as foreskin-derived induced pluripotent stem cells, was broadened by coculture with AT-MSCs. The authors concluded that despite the limited evidence, the current data indicate that the use of MSCs may provide beneficial effects for periodontal regeneration. Furthermore, the implantation of local MSCs was not associated with adverse effects and improved the regenerative outcomes of periodontal defects treated with bone substitutes, while periodontal ligament-derived MSCs consistently promoted increased periodontal ligament and cementum regeneration [19]. The various degrees of success of MSCs in periodontal regeneration might not only be related to the heterogeneity of the cells used but the effect of exosomes as well. Bright et al. in a systematic review of the use of periodontal ligament-derived MSCs for periodontal regeneration came to a similar conclusion [20]. Overall, regardless of the defect type and animal model used, periodontal ligament stem cell implantation results in beneficial outcomes for periodontal regeneration.

It is more and more recognized that medicinal signaling realized by secreted bioactive compounds and exosomes induces regenerative processes. In fact, Caplan [21] suggested that the name of MSCs should be changed to medicinal signaling cells to more accurately reflect the fact that these cells home in on sites of injury or disease. Specifically, periodontal tissue regeneration was found to be promoted by secretomes in CM from human MSCs [22]. The addition of a cytosine cocktail composed of insulin-like growth factor-1, vascular endothelial growth factor-A, and transforming growth factor-*β*1 gave a similar effect in periodontal tissue regeneration in dogs [23]. Furthermore, the osteogenic differentiation potential of human DP-MSCs for bone regeneration has been effectively proven using a model of calvarial defects in rabbits employing DP-MSCs/poly(*ε*-caprolactone)-biphasic calcium phosphate scaffold constructs [24]. Huang et al. evaluated the potential of DP-MSC exosomes to induce odontogenic differentiation of the naïve human dental pulp stem cells and of BM-MSCs and found that the exosomes bound to matrix proteins such as a type I collagen were endocytosed by both DP-MSCs and BM-MSCs, triggering an increase in the expression of genes required for odontogenic differentiation. Thus, it has been concluded that DP-MSC-specific exosomes can trigger lineage-specific differentiation of stem cells [25].

## 5. Human Tooth Revitalization Mediated by MSCs

There are attempts to innovate classical endodontic therapies consisting of disinfecting and then sealing the endodontic space with dental stem cells. Recent findings suggest that regenerating fully functional pulp tissue may be an ideal therapeutic solution to maintaining a tooth defense system by detecting and helping to manage future injuries [26]. Future prospects of tissue engineering approach to dentin/pulp regenerative therapy were recently comprehensively reviewed [27]. DP-MSC-mediated dental pulp regeneration is considered a promising method for the treatment of deep caries with pulpitis. Based on the results of a pilot clinical study on the feasibility of autologous transplantation of DP-MSCs in pulpectomized teeth, Nakashima et al. concluded that human DP-MSCs are safe and efficacious for complete pulp regeneration in humans [28]. Furthermore, studies devoted to determining the role that root canal disinfection plays in regenerative endodontic treatments have shown that a biocompatible irrigant is acceptable in low concentrations [29, 30]. In the context of the involvement of exosomes from MSCs in the regenerative process, it is likely that a future approach for pulp regeneration might consist of conditioned medium and/or exosomes bound to a suitable scaffold (perhaps collagen membrane). Also, cell exclusion might lead to a very practical therapeutic tool composed of allogeneic material originating from DP-MSCs of young donors. Moreover, DP-MSCs have been found to be useful for human cleft lip and palate reconstruction [31, 32].

## 6. Whole Tooth Regeneration Approaches

A long-term goal of dentistry is to create functional biomimetic tooth buds for eventual tooth replacement in humans. The tooth is an ectodermal organ composed of soft connective tissues, a distinctive hard tissue, and additional tissues like peripheral nerve fibers and blood vessels. Tooth development is regulated by mutual interactions of mesenchymal and epithelial cells, and considering our present knowledge about exosomes as factors capable of transferring bioinformation between cells, their involvement in this natural process seems likely.

To recapitulate organogenesis, it is required to have compartmentalization of epithelial and mesenchymal cells at a high enough cell density to mimic multicellular assembly conditions and epithelial-mesenchymal interactions leading to a bioengineered tooth germ. The tooth germ is capable of generating a structurally correct tooth in vitro and erupts successfully with the correct tooth structure when transplanted into the oral cavity. For example, whole-tooth restoration has been demonstrated both in a murine tooth-loss model [33] and when utilizing autologous bioengineered tooth germ transplantation in a postnatal canine model [34]. Zhang et al. were the first to show that decellularized tooth buds (dTBs) created from unerupted porcine tooth buds can be used to guide reseeded dental cell differentiation to form completely bioengineered teeth in a minipig model. Furthermore, the formation of organized, bioengineered teeth of comparable size to natural teeth has been observed 6 months after implantation of recellularized dTBs seeded with porcine dental epithelial cells, human dental pulp cells, and human umbilical vein endothelial cells. It is thought that intercellular communication between the involved cells by means of nanovesicles was most likely responsible for the successful regeneration process [35].

Dental pulp stem cells are obviously the most proper source of MSCs for tooth regeneration. Although these cells demonstrate high growth potential, the small size of dental pulp tissue is currently a limiting factor for the expansion of the large cell number needed for regenerative dentistry. Hung et al. tested the possibility of using AT-MSCs, which are an easier cell source than DP-MSCs for tooth regeneration. They showed that by using strictly controlled, side-by-side comparisons between the two types of stem cells that the expression patterns of gene markers were very similar. Then, they demonstrated that both implants of AT-MSCs and DP-MSCs were able to grow self-assembled new teeth in adult rabbit extraction sockets [36]. This finding creates general notion that AT-MSCs, one of the richest sources for adult stem cells in mammals, can be a very versatile and useful tool for regenerative medicine.

## 7. DP-MSC Senescence

Mesenchymal stem cell senescence is an adverse factor from the perspective of any cell-based therapies. Several studies have shown that senescence impairs the proliferation and differentiation potentials of DP-MSCs and that donor age is an important factor that affects their use for tooth regeneration. The age-associated changes of DP-MSC, determined by comparing isolated cells from young and aged dog teeth, were attributed to a decrease in the regenerative potential of resident stem cells [37]. However, pretreatment of MSCs with macrophage migration inhibitory factor has been found to rejuvenate endogenous bone marrow-MSCs in aged individuals [37]. On the molecular level, the analysis revealed that 304 mRNAs were differentially expressed, including 247 upregulated and 57 downregulated genes in DP-MSCs from young donors compared to aged subjects. Furthermore, in DP-MSCs from young donors, numerous long noncoding RNAs were either significantly up- or downregulated compared to DP-MSCs from aged donors [38]. Roforth et al. examined gene expression and epigenetic changes in the level of DNA methylation patterns from highly enriched BM-MSC populations taken from young versus aged women. They identified 279 differentially expressed genes involved in both protein synthesis and degradation pathways between the young and old subjects [39]. Huang et al. stressed the importance of the microenvironment for conversion of DP-MSCs into odontoblast-like cells, showing that DP-MSCs seeded onto dentin surfaces result in the formation of cells with odontoblastic morphologies [40]. Also, Zhai et al. concluded from experiments with DP-MSCs damaged by hydroxyurea in vitro that DP-MSCs from young donors are more resistant to apoptosis and exhibit increased nonhomologous end joining DNA-repair activity compared to aged donors [41]. Cumulatively, it can be concluded that for the purpose of regenerative medicine, deciduous tooth pulp-derived cells are the best choice, not only because of their hardiness but also due to their robust expansion by cultivation in vitro.

## 8. MSCs Release Complex Secretome and Nanoparticles

It has been well documented that MSCs release rich secretomes containing massive amounts of cytokines, chemokines, and growth factors, together with extracellular nanoparticles. The most important nanoparticles in the medium conditioned by MSCs are exosomes. Exosomes are extracellular vesicles of 30–120 nm in diameter secreted by cells that act as messengers by communicating with other cells and carry intracellular sorted cargo that is encapsulated in a lipid bilayer-bound vesicle. While all cells purge intracellular waste by a release of exosomes, those formed in the cell by endocytosis include important informative molecules, like microRNAs, mRNAs, and overexpressed proteins in their cargo. A cell-derived lipoprotein coat of exosomes protects their cargo from degradation in the systemic circulation and determines their tropism. After internalization into recipient cells, MSC-exosomes have the ability to change them to specifically differentiated cells. This regenerative process starts by paracrine action of MSC secretome on intrinsic native cells of the recipient.

### 8.1. Exosomes as Diagnostic Markers and Targeted Therapeutic Tools

Exosomes are released from all cells, and their internal cargo reflects the cell's status. Additionally, exosomes are capable of intercellular communication. For instance, exosomes derived from tumor cells signal cellular aggressiveness and carry information about resistance to previously used therapeutic treatments (chemotherapy, radiotherapy), thereby representing a useful marker for the selection of personally suited therapies, such as when tumor cell-derived exosomes were shown to determine organotropic metastasis, creating a premetastatic niche [42]. Consequently, exosomes can serve as important diagnostic and prognostic markers as they are able to penetrate cells and after internalization, they change the expression of cellular genes of the recipient cells. Furthermore, when MSCs are genetically modified to express therapeutic gene(s), the exosomes released contain mRNA of these genes. As a result, MSCs exposed to drugs include them in their exosome's cargo. Such exosomes represent a highly attractive delivery vehicle for any gene we wish to be expressed in recipient cells [13] and/or for therapeutic drugs [43].

Exosomes were originally recognized as nanoparticles secreting cellular waste [44]. However, we have shown that the exosomes released from the DP-MSCs labelled with iron oxide nanoparticles are a tumor-targeting tool for hyperthermia therapy mediated by an alternating magnetic field [45]. Additionally, Stremersch et al. reviewed the potential of extracellular vesicles as future biomarkers, drug delivery vehicles, and potential cell-free vaccines. Furthermore, exosomes may be useful as a diagnostic tool as the composition of their cargo reflects the physiological status of the producing cell and tissue [46].

## 9. MSCs and Their Secreted Components in Regenerative Medicine

Human MSCs are widely available from various tissues and have been proposed as a promising cell replacement therapy for maladies of the geriatric population. Therapy for neurodegenerative diseases such as Parkinson's disease, Alzheimer's disease, amyotrophic lateral sclerosis, multiple sclerosis, spinal muscular atrophy, and Huntington's disease currently lack effective treatments. There is an increasing amount of evidence that MSC-conditioned medium containing exosomes, or the exosomes themselves, reiterates the regenerative induction capability of particular MSCs and retains the homing properties of their parent cells. The beneficial effect of exosomes derived from dental mesenchymal/stromal cells has been shown in neurodegenerative diseases, peripheral neural regeneration, myocardial infarction, focal cerebral ischemia, stimulation of angiogenesis, neurogenesis in the ischemic rat brain, acute lung injury, suppression of acute inflammation, and syndromes connected with immunosuppression or autoimmunity, and the list is likely not final.

The importance of the paracrine action of components released from MSCs has been recognized, but the exact mechanisms behind these effects are presently unknown. Rich MSC secretome and a number of bioinformative compounds in an exosome's cargo are involved, but how the damaged cells choose the competent ones remains to be elucidated. Nevertheless, it is not exceptional in medicine to use therapeutically beneficial tools without exact knowledge of their actions. Additionally, MSC-derived exosomes have multiple advantages over cell therapy, because exosomes are stable in the systemic circulation without losing function, and in comparison to stem cells, they exhibit a superior safety profile.

Potential, broad therapeutic applications of microvesicles, especially exosomes, was overviewed in the position paper of the International Society for Extracellular Vesicles [47]. In Parkinson's disease, it was initially believed that MSC transplantation could replace neuronal loss by differentiation of administered cells. With the recognition of the paracrine action of MSC-mediated regenerative processes, exosomes became an attractive choice for the treatment of neurodegeneration. Out of various tissue-derived MSCs, MSCs derived from dental pulp and/or from exfoliated deciduous teeth are of particular interest as they are of embryonic origin and from the ectoderm layer of the neural crest. As such, they have been intensively studied to exploit their therapeutic potential for neurodegenerative diseases, revealing that exosomes released from DP-MSCs have the potential for treating neurodegenerative diseases.

Many preclinical and clinical studies have been published showing the positive effect of cell transplantation in Parkinson's disease and peripheral neural regeneration [48]. DP-MSC exosomes, which are capable of penetrating the blood-brain barrier when administrated systematically, could slow down or replace neuronal loss in Parkinson's disease [49]. For example, Salama et al. showed that in a mouse model of Parkinson's disease, intranasal stem cell administration alleviated disease symptoms [50]. The study validated the use intranasal administration of exosomes as an easy, cheap, and safe alternative route for treatment of neurodegenerative disorders.

## 10. General Therapeutic Effects of DP-MSC-Derived Exosomes

Exosomes derived from DP-MSCs have been shown to suppress experimentally induced acute inflammation in mice and reduced induced edema to similar levels seen with prednisolone as a positive control. However, the effects of prednisolone were more prominent at the early stages, while exosomes exerted their suppressive effects gradually and at later time points [51]. The therapeutic potential of dental pulp MSC-derived conditioned medium (CM) was found to be similar to the injection of live cells in a mouse model of neuropathic pain, producing a powerful and long-lasting antinociceptive effect [52].

Recent developments in peripheral nerve regeneration have shown that human gingiva-derived mesenchymal stem cells are easily induced into neural progenitor-like cells. These cells, when transplanted to the injury site, promoted recovery in the crush-injury model of rat sciatic nerve. The molecular mechanism behind this regeneration was the expression of the antagonistic myelination regulators c-Jun and Krox-20/EGR2 in Schwann cells [53]. Very recently, in a preclinical evaluation, a similar positive effect against neuropathic pain with CM derived from bone marrow MSCs was reported [54]. The immunomodulatory and regenerative properties of human DP-MSCs are reflected in the expression of embryonic stem cell markers like SKIL, MEIS1, and JARID2. These markers, as well as cell proliferation markers, were shown not to be influenced by the total number of in vitro passages. This finding supports the application of dental-derived MSCs in stem cell therapy-based clinical trials [55].

Song et al. compared the therapeutic effects of transplanted human DP-MSCs and human BM-MSCs in a stroke model of rats. Either human DP-MSCs or BM-MSCs were intravenously injected into rats after middle cerebral artery occlusion and showed improved functional recovery and reduced infarct volume versus control rats. Furthermore, the DP-MSC-treated group showed greater reduction in infarct volume than BP-MSCs [56]. MSC exosomes were also found to induce long-term neuroprotection, while promoting neuroregeneration and neurological recovery in a rodent stroke model [57]. The broad therapeutic effects of MSC exosomes being capable of inducing long-term postischemic neuroprotection, sustained neurological recovery, neurogenesis, and angiogenesis were recently comprehensively reviewed. The use of MSC exosomes in regenerative medicine is clearly advantageous compared with the administration of cells [58]. Furthermore, Sarko and McKinney recently reviewed the origin of exosomes and their therapeutic potential for neurodegenerative diseases [59]. CM derived from human stem cell exfoliated SHEDs was tested as a treatment for experimentally induced acute respiratory distress syndrome in mice, and a single intravenous administration of either cells or SHED-CM attenuated lung injury and weight loss, while improving their survival rate. Taken as a whole, the advantage of CM or exosome administration is obvious [60].

## 11. Tumor Tropism of DP-MSCs and Exosomes

MSCs recognize a tumor as a nonhealing wound [61]. As such, the MSCs migrate to it and frequently become a part of tumor stroma. The tumor tropism of MSCs engineered to express suicide gene yeast fused cytosine deaminase uracil phosphoribosyl transferase (yCD::UPRT) was found to be sustained [62]. All types of MSCs release rich secretome composed of low molecular weight components and exosomes [63]. Furthermore, the exosomes released from the DP-MSCs transduced with the suicide gene yCD::UPRT contain its mRNA in their cargo. These exosomes penetrate easily and integrate human tumor cells and, in the presence of prodrug 5-fluorocytosine, cause apoptosis in tumor cells [64]. DP-MSCs possess brain tumor tropism, and the exosomes released from them mirror their tissue-specified homing. We have shown that human DP-MSCs labelled with iron oxide nanoparticles sustain their tumor tropism in vivo as demonstrated by the ability of intranasally administered cells to migrate and specifically engraft orthotropic glioblastoma xenografts in rats [13].

Diffuse intrinsic pontine gliomas (DIPGs) are fatal tumors found in the pons of young children and do not respond to standard therapies like radiotherapy or chemotherapy. However, nanomedicine may offer novel therapeutic options for the treatment of DIPGs. Multiple nanoparticle formulations capable of traversing the blood-brain barrier into the pons like Qdots, gold nanoparticles, dendrimers, and liposomes have been studied as a potential tool for therapeutic intervention [65]. For example, effective treatment of rat glioblastoma with the prodrug gene therapy mediated by human AT-MSCs engineered to express a suicide gene was likely the consequence of the action of exosomes [66]. We believe that these exosomes can be potentially used for targeted therapy of DIPG.
